# A New Method to Compare Statistical Tree Growth Curves: The PL-GMANOVA Model and Its Application with Dendrochronological Data

**DOI:** 10.1371/journal.pone.0112396

**Published:** 2014-11-17

**Authors:** Martin Ricker, Víctor M. Peña Ramírez, Dietrich von Rosen

**Affiliations:** 1 Departamento de Botánica, Instituto de Biología, Universidad Nacional Autónoma de México (UNAM), México D.F., Mexico; 2 Posgrado en Ciencias Biológicas, Universidad Nacional Autónoma de México (UNAM), México D.F., Mexico; 3 Department of Energy and Technology, Swedish University of Agricultural Sciences, Uppsala, Sweden; University of Cambridge, United Kingdom

## Abstract

Growth curves are monotonically increasing functions that measure repeatedly the same subjects over time. The classical growth curve model in the statistical literature is the Generalized Multivariate Analysis of Variance (GMANOVA) model. In order to model the tree trunk radius (*r*) over time (*t*) of trees on different sites, GMANOVA is combined here with the adapted PL regression model *Q* = *A*·*T*+*E*, where for 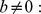 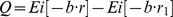 and for 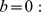 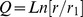, *A* =  initial relative growth to be estimated, 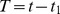, and *E* is an error term for each tree and time point. Furthermore, *Ei*[–*b*·*r*]  = 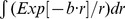, 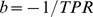, with *TPR* being the turning point radius in a sigmoid curve, and 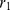 at 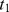 is an estimated calibrating time-radius point. Advantages of the approach are that growth rates can be compared among growth curves with different turning point radiuses and different starting points, hidden outliers are easily detectable, the method is statistically robust, and heteroscedasticity of the residuals among time points is allowed. The model was implemented with dendrochronological data of 235 *Pinus montezumae* trees on ten Mexican volcano sites to calculate comparison intervals for the estimated initial relative growth 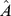. One site (at the Popocatépetl volcano) stood out, with 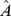 being 3.9 times the value of the site with the slowest-growing trees. Calculating variance components for the initial relative growth, 34% of the growth variation was found among sites, 31% among trees, and 35% over time. Without the Popocatépetl site, the numbers changed to 7%, 42%, and 51%. Further explanation of differences in growth would need to focus on factors that vary within sites and over time.

## Introduction

Growth curves are monotonically increasing functions that measure repeatedly the same subjects over time. Statistical growth curve modeling of trees continues to be basic to forest science (Chapter 6 in [1]): Where are the best sites for production of timber or other products from trees? What factors explain growth variation? Can those factors be influenced by management? What is the commercial value of a timber plantation, for example in 30 years? How long does it take to reforest a given site?

Considering the relationship between quantity and time, there are at least three fundamental variables involved in determining the mathematical form of a growth function: the curve's shape, its growth rate, and its starting quantity (or more generally the curve's positioning quantity). Many different functions have been used or at least suggested [2], [3], but none is accurate over the whole lifespan of a tree; for that purpose, the curve has generally to be segmented with some form of spline modeling. An important reason is that tree growth curves vary not only according to the species, but also in response to environmental circumstances that may suddenly change. For example, a tree may be suppressed for a time by competition, and then released by a gap opening, which may result in two joined sigmoid growth curves.

Ricker and del Río [4] presented a new tree growth model that employs piecewise linear regression to relate logarithmic relative growth of the tree trunk diameter with the diameter itself. The objective was to apply it for tropical trees, for which annual tree growth rings could not be identified. This model, which they called PL (piecewise linear) model, has some interesting mathematical properties, such as straightforward interpretation of the regression coefficients and high flexibility to model sigmoid, exponential or over-exponential growth.

The second model employed here is the GMANOVA (generalized multivariate analysis-of-variance) model. It is also called “Potthoff-Roy model”, according to its formulation by Potthoff and Roy in 1964 [5], or the “classical growth curve model of statistics”, because many articles about it have been published in journals of statistics (for reviews see [6]–[13]). The term “GMANOVA model” describes the model much better than the in the statistics literature widely used term “growth curve model”, because many other approaches to analyze growth curves exist: see, for example, [14] in the context of plant growth analysis, [1] or [15] in forest science, [16] in econometrics, and [17] in the social sciences. To our knowledge, the GMANOVA model has never entered the literature of tree growth modeling, possibly because of its relatively mathematical and theoretical treatment in the statistics literature.

Here we take a new approach to employ the PL model in combination with the GMANOVA model. We call the combination the PL-GMANOVA model (pronounce “P”-“L”-“G”-“MANOVA”). After developing the model in the methods section, we apply it to dendrochronological data of 235 trees on ten different Mexican volcano sites.

## Methods

The basic idea is to use the growth function of the PL model, which has some interesting mathematical features, together with the GMANOVA model, which compares growth statistically among different groups. First, the PL model is used individually for each tree with nonlinear regression, in order to reduce the growth curve to three regression coefficients. Then, two of those three regression coefficients per tree are employed in a linearized version of the PL model's growth curve formula, which enters the linear GMANOVA model to compare relative growth of individual trees, grouped for sites.

### The PL Model

The PL (piecewise linear) model was originally published by Ricker and del Río in 2004 [4], to relate logarithmic relative growth with quantity in one or several piecewise linear segments when tree ages are unknown. The basic formula for a single segment is 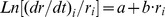, where 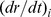 refers to the (instantaneous) annual increment of tree trunk radius 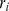. This formula can be converted mathematically into the following equation to model the time it takes to grow from tree trunk radius 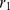 at time 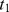 to 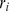 at time 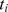 of data point *i* (page 217 in [4]):
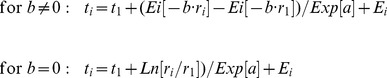
(1)


**Figure 1:**
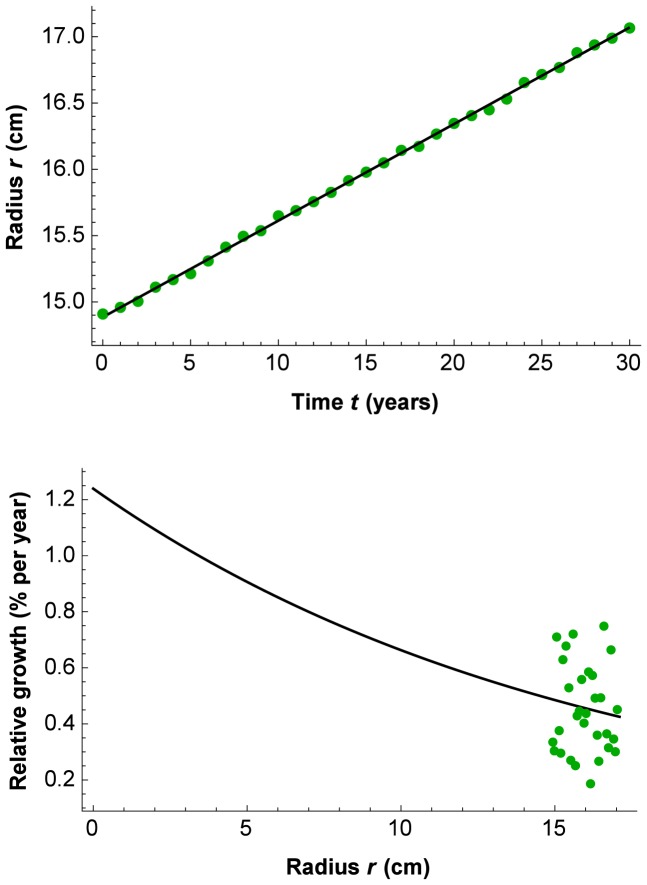
Growth of tree #52 from the Chichinautzin site. *Above*: Regression line 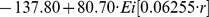 from nonlinear regression with (1) and fixed 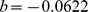 cm^−1^ to find 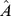 = 1.24% per year and radius 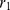 = 15.98 cm at time 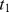 = 15 years (1989; *R* ^2^ = 0.9998). *Below*: Initial relative growth (here 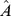 = 1.24% per year) represents a standardized measure for comparing relative growth (*dr*/*dt*)/*r*, even though the data points may be at different radiuses: The mean growth path is projected backward to a radius infinitesimally close to zero; when *b*<0, *b* determines the change of relative growth with increasing radius as a negative exponential function (Relative growth  = *A*·*Exp*[*b*·*r*]). Note that the graph does not show an extrapolation, but a standardized characterization of relative growth at the *Y*-intercept.

Here, 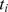 is time, age, or date of data point *i*; 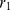 at 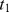 is the tree trunk radius at given time or age for positioning the overall growth curve (either 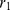 or 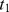 can be used as regression coefficient); *Ei* is the exponential integral *Ei*[–*b*·*r*]  = 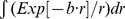; *Exp* refers to the exponential function; *b* is a regression coefficient, representing the negative inverse of the turning point radius (*TPR*) in a sigmoid curve; 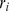 is the tree trunk radius at time 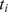 of data point *i*; *a* is a regression coefficient, representing initial logarithmic relative growth, with *Exp*[*a*]  = *A* =  initial relative growth (see Figure 1 why it is called “initial”); 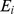 is the error term for data point *i*; and *Ln* refers to the natural logarithm.

The term *Ei*[–*b*·*r*] represents the integral of *Exp*[–*b*·*r*]/*r* over *r*, and is dimensionless (see page 164 in [18] or Appendix 3 in [4] for additional references and a relatively easy method for numerical calculation). For *b* = 0, the term *Exp*[–*b*·*r*]/*r* simplifies to 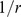, and its integral is not *Ei*[–*b*·*r*] anymore, but simply *Ln*[*r*].

Equation (1) is employed here directly in nonlinear regression. The growth function *t* = 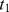
+ (*Ei*[–*b*·*r*] – *Ei*[–*b*·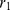])/*Exp*[*a*], however, cannot be solved in closed form for *r* as a function of *t* (as one would ideally like); for applying regression analysis one has to use the inverse function, with *t* being the dependent variable (with the corresponding residuals), for given *r* as the independent variable. Therefore, we model time as a function of radius, rather radius as a function of time.

We introduce here both *a* and *Exp*[*a*]  = *A*, because *a* is a coefficient in [4] and works numerically better for nonlinear regression with (1); however, subsequently our whole discussion will focus on the estimated initial relative growth 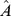, determined directly by GMANOVA. The parameter *A* can also be more intuitively interpreted and expressed in percent per year (by multiplying with 100%). Figure 1 bottom illustrates that initial relative growth represents a standardized parameter, when relative growth changes with radius.

Note that in Figure 1 bottom, the PL model relates relative growth with radius, not time. There is a high correlation between time (age) and radius for data in the case of single trees. When calculating with our data the 235 correlation coefficients between time and radius for each 30-years segment, these range from 0.919 to 0.9996, the median being 0.994. On the other hand, when not distinguishing individual trees, the correlation coefficient is 0.05, i.e., not significantly different from zero. Consequently, tree radius is a good proxy variable for tree age in individual trees, but does not serve to infer the age of individual trees in a group of trees, i.e., a small tree can be old and a large tree can be young. Consequently, (1) with nonlinear regression should be applied to individual trees. Figure 1 (top) shows also in the example that autocorrelation of the residuals is of little concern, because the residuals are very small in relation to the estimated radius.

Below, we will convert the nonlinear relationship in (1) into a linear relationship that can be used in the GMANOVA model.

### The GMANOVA Model

The generalized multivariate analysis of variance (GMANOVA) model attempts to explain variation of the dependent variables (e.g., tree trunk radius) for different individuals (e.g., trees) in different groups (e.g., trees per site) at different time points (e.g., years). One can also analyze a single group (page 308 in [12]). Originally designed for observations taken always at the same time points and without missing data, some authors have also considered the more complicated cases of irregular time points [19] or incomplete data [20], though this will not be applied here. The GMANOVA is fundamentally a multidimensional, linear matrix model. We use the following notations for matrices and their operators: ***X***: *r* 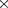 *c* is a matrix (therefore in bold) of *r* rows and *c* columns; 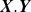 multiplies matrices (or vectors) ***X***: *i* 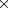 *j* and ***Y***: *j* 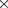 *h*; 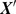 is the transpose of matrix ***X***; 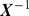 is the inverse of a nonsingular matrix ***X***; ***X***∶***Y*** is the Kronecker product between matrices ***X*** and ***Y***; and 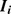 is an identity matrix with *i* rows as well as *i* columns. Furthermore, it is useful to define the “mean” as the expected value or “centre of mass” (location) of a probability distribution, or more formally as the sum of all possible values of a random variable, each multiplied by its probability. On the other hand, “average” refers to the arithmetic mean. The term “error” refers to the difference between the parametric mean of the model and the true value of an observation, while the term “residual” refers to the difference between the estimated mean of the model and the measured value of an observation.

An exposition with derivations of several formula in this section can be found in [9] and Chapter 4 in [13], though the notation and some formulas are adapted here; also, for example (8) is new in our context, and some relationships are not stated explicitly in the literature. The basic formula of the GMANOVA model is the following equation:
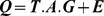



The matrix ***Q***: *p* 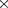 *n* is the data matrix of quantities to be explained for *p* time points (rows) and all *n* individuals in all groups combined (columns). Each individual (here tree) is represented with a complete data curve over all *p* time points. The letter “*Q*“ stands for “quantity” (here *Ei*[–*b*·*r*] – *Ei*[–*b*·*r*], as explained in the next section about the PL-GMANOVA model). How to arrange the data points in the matrix ***Q*** depends on the design matrix ***G***. The matrix ***T***: *p* 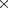
1 is the within-individuals design matrix for *p* time points (and here *q* = 1 polynomial term for determining only the slope of a regression line through the origin). The letter “*T*” stands for “time”, being here data points of the time intervals 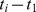. The vector ***A***: 1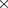 *k* contains the regression coefficients 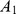 to 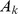 (here mean initial relative growth per site) for the *k* treatment groups (here sites). The matrix ***G***: *k* 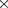 *n* is the among-individuals design matrix for *k* treatment groups (rows) and *n* individuals (columns). The letter “*G*” stands for “groups” (here of trees on different volcano sites). The error matrix ***E***: *p* 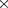 *n* contains columns that are independent *p*-variate normal with mean vector **0** and commonly unknown covariance matrix among the *p* time points **Σ**>0; **Σ** (“sigma”) is estimated with (4). The letter “*E*“ stands for “error” of the ***Q*** data. The matrix **Σ** is assumed to be the same for each individual of a given group. In other words, the errors may be heteroscedastic over time, but should be homoscedastic among groups at a given time point. Furthermore, the covariance among trees over time is assumed to be zero, i.e., their growth paths are not linearly dependent on each other.

The mathematical form of the growth curve model that has been used in the statistical literature, is a polynomial of a degree that provides a good fit to model the mean growth, such as tree trunk radius as a function of time for a given group (for example, of degree two: 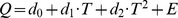, with 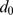 to 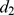 being regression coefficients). Matrix ***T*** has to be designed according to the chosen polynomial degree; in our application, we use a straight line through the origin, which simplifies the model to 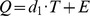. Some of the following matrix formulas also simplify a little bit, and some of the matrices, such as ***T*** and ***A***, reduce to vectors. To get a more intuitive idea what the potentially large matrices ***Q***, ***T***, ***G***, and ***A*** look like, an example is given for *p* = 6 time points and *n* = 12 trees in *k* = 3 groups:
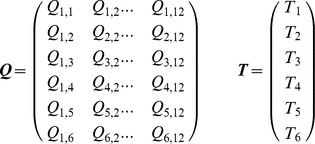


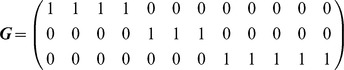



With the data in ***Q*** and ***T***, and the groups defined by ***G***, the to-be-estimated parameters are 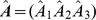.

There are several methods of estimation available for the GMANOVA model, the most common ones being maximum likelihood estimation and the least squares estimation. Both estimators refer here to the mean initial relative growth 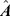 per site, as explained in Figure 1. The first step for maximum likelihood estimation is to calculate the sum-of-squares matrix among *p* time points:
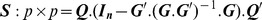



It is assumed that 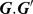 is invertible. The sum-of-squares matrix ***S*** has to be non-singular, i.e., reflect variation rather than linear dependencies. Its determinant should therefore differ from zero. Note, however, that a well-conditioned matrix can have a very small determinant (page 82 in [21]). If the matrix is so ill-conditioned that the determinant of ***S*** is numerically indistinguishable from zero (which depends in turn on the software used), one can use the (generalized) Moore-Penrose inverse of ***S*** in the subsequent formulas. This can happen when the measurements of *Q* at *T* contain a lot of repetitive information. The Moore-Penrose inverse solves technically the estimation of the inverse in that case, while it is identical to the standard inverse otherwise.

The Maximum Likelihood Estimator 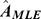 for the *k* treatment groups is calculated as:
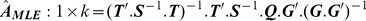
(2)


Alternatively, the Least Squares Estimator equals:
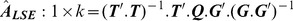
(3)


Pan and Fang [9] refer to 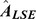 as the “generalized least squares estimator”, because it minimizes the trace of 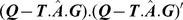, but a generalized least squares estimator is usually a special type of weighted estimator; consequently the term “least squares estimator” seems more appropriate for 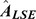.

The difference between the maximum likelihood estimator and the least squares estimator is the weighting by the sum-of-squares matrix ***S*** in case of the maximum likelihood estimator. Only for certain covariance structures, the two estimators do give the same results (pages 77–78 in [9]). Usually, the numerical results are rather similar. Both estimators are unbiased. The estimator 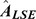 is normally distributed if ***Q*** is normally distributed, while this is not the case for 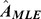, which has an unknown distribution that is never completely normal for a finite number of observations (because ***S*** is a random matrix). However, 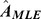 is asymptotically (with increasing number *n* of individuals) the best estimator, in terms of having the lowest possible variance, and its distribution is also asymptotically normal. The literature has largely focused on the maximum likelihood estimator, also for statistical inference [22]. In our application, the maximum likelihood estimator is much more sensitive to non-normal data than the least squares estimator, which has to do with the mirror image of the data in the I. and III. quadrants (in the Cartesian coordinate system). In forestry data, the presence of outliers and mixed distributions is common, causing such non-normality, and the maximum likelihood estimator gives unreasonable results in that case. Therefore, we use first the least squares estimator to detect outliers, and subsequently the maximum likelihood estimator of the (potentially transformed) data without outliers.

The estimated covariance (or dispersion) matrix 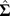 reflects the data variation; the square root of its leading-diagonal values is the standard deviation at each time points. The formula is the same for both estimators (with 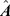 depending on the estimator):
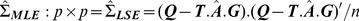
(4)


Both estimators require a minimum of independent observations *n*: The maximum likelihood estimator requires 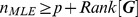, in order to avoid a singular sum-of-squares matrix ***S***, and being able to take its inverse in (2). The least squares estimator requires 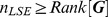, in order to avoid singularity of 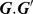, and being able to take its inverse in (3). For example, with 10 groups and 30 time points, 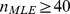 and 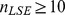. In the case of the least squares estimator it makes sense that with 10 groups one needs at each time point at least 10 independent observations, one per group, though the covariance matrix of (4) cannot be estimated with one observation per group. With 40 independent observations at each time point (on average four per group), the maximum likelihood estimator is much more demanding to get the estimator for ***A***, but the same number of observations is required for estimating the covariance matrix of the least squares estimator. For that reason, the growth curve model is generally applied for short time series, where “short” in this context means 4 to 40 time points as a reasonable range. For longer time series with insufficient *n*, one needs to put a structure (model) on the covariance matrix (see [9]), though this has not to be done here.

The estimated covariance (or dispersion) matrix of the mean estimator 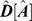 reflects the variation of 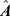, and will serve us to calculate the standard errors of the mean initial relative growth parameters per site as:
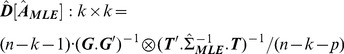
(5)


Note that 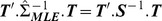, which shows that the variances and covariances are independent of the mean estimator. The corresponding formula for the least squares estimator is:
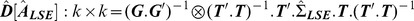



Even though 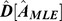 presents asymptotically the lower variance, one cannot generalize for a given finite data set which of the two estimators has lower variance. The diagonal elements correspond to the error mean squares in analysis of variance, and the vector of estimated standard errors of 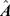 can be calculated for both estimators as follows:
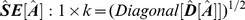
(6)


*Diagonal* refers to the values on the leading diagonal of matrix 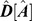, and the square root is taken of each value. The standard errors vary only according to differences in the number of data points per group, and in a completely balanced design such standard errors are the same for each group.

An assumption of the GMANOVA model is that the errors at each time point and within each group are normally distributed. In particular skewness of the errors causes a poor prediction of the mean path of the growth curve. After deleting obvious outliers (see below), a transformation may still be needed. A both-sides power transformation works well for the PL-GMANOVA model (see the section on transformation below for details).

Finally, we are interested to calculate residuals of the unexplained variance of ***Q***, for evaluating the model fit. The usual raw residuals (observed minus predicted values) are:
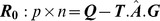
(7)


These are the raw residuals around the regression line that should be symmetrically distributed (only asymptotically normally in maximum likelihood estimation), or the data should be transformed. In addition to checking normality, they can be used to calculate the coefficient of determination for the model fit. The total variance of the data is the variance of all data entries in ***Q*** = 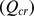, where *c* is the column index and *r* is the row index. The unexplained variance is the sample variance of all entries in 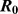 = 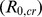. Taking one minus the computational formula of the unexplained variance, dividing by the total variance, and simplifying, results in:
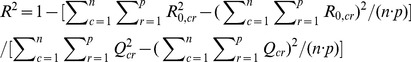
(8)


While heteroscedasticity of the raw residuals in 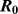 is allowed among time points, homoscedasticity is an assumption among groups for a given time point *T*. This can be analyzed by calculating the variance of the residuals for each group, calculated with the usual sample variance formula. Heteroscedasticty among groups affects the standard errors of the estimates of *A*, and consequently statistical inference.

Finally, for the maximum likelihood estimator von Rosen [23] derived three subtypes of residuals (components of the raw residuals) that serve to analyze the model fit:
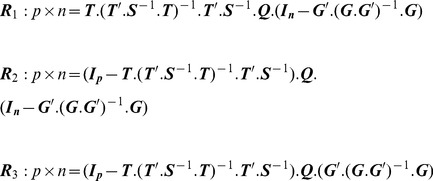
(9a – c)


The sum 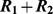 is the matrix of the residuals between a group's observations (*Q*) and the group's arithmetic mean; 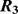 is the matrix with the residuals of the difference between the group's arithmetic mean and the estimated regression model 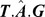; the sum 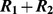 could be calculated directly as 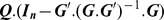, but it is recommended to calculate 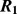 and 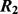 separately, because elements in these matrices may appear with opposite signs and of the same size, causing their sums to be very close to zero (page 131 in [23]). The relationship between the different types of residuals for an individual residual is 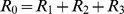; furthermore, for the matrices the relationship 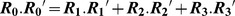 holds.

Ideally the regression line should go through the arithmetic means of the group's observations (*Q*) at each time point (*T*). The residuals 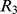 represent deviation from that ideal situation. As a general criterion of a satisfactory model fit we like to see that the absolute values of the residuals 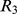 are smaller than the absolute values of 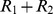, i.e., the observation's dispersion due to factors other than a deficient model fit. If 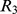 residuals dominate the model, then it is more important to look for a better model than trying to explain nature's growth variation.

We use the absolute values (*Abs*), because negative residuals of one type and positive residuals of another type would mask each other. The absolute values of the residuals, however, are not normally distributed, but at best represent the positive half of a normal distribution. Therefore one can calculate the ratio *Median*[*All Abs*[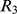]]/*Median*[*All Abs*[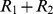]], where *All* means *k*·*p* residuals of 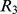 (after deleting repeated 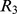 for each group and time point), and *n*·*p* residuals of 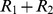. The ratio of the medians should be smaller than 1, and is 0 with a perfect model. One can test the (un)equality of the two medians with a modified, non-parametric Mann-Whitney *U*-test [24].

Autocorrelations of the means of *Q* among time points in a given group show up as an imperfect model. Left-over skewness after a global transformation may cause such autocorrelation. The above criterion is useful to decide if autocorrelation is too severe to be acceptable.

### Construction of the new model: Combination of the PL Model and the GMANOVA model into the PL-GMANOVA model

Using polynomials in GMANOVA has been a convenient way to model nonlinear growth curves with a model that is linear in its parameters, when not knowing anything about the functional relationship between time and the modeled variable. Using here a functional form of the PL model in the GMANOVA model is a different way to model nonlinear growth curves with a linear model, but with two major advantages:

1. The estimated vector *A* has a clear interpretation, being the initial relative growth parameters for the *k* groups (see Figure 1).
2. The 2–3 polynomial terms that are typically applied in GMANOVA reduce to a single term, i.e., a straight line through the origin, simplifying the model fit and its interpretation.

In order to combine the GMANOVA model with the PL model, define:
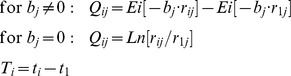



Then, (1) can be transformed into the following linear relationship:
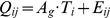
(10)


The variable 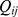 represents a dimensionless quantity at time point *i* for tree *j*, and each tree *j* pertains to a group *g*; 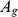 is the mean initial relative growth of the trees in group *g*; 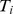 is the time (interval) point *i*; and 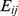 is the error term for untransformed data of tree *j* at time point *i*.

The quantities *Q* = *Ei*[–*b*·*r*] – *Ei*[–*b*·*r*_1_] are the data points to be explained by time periods and tree groups per site; *Q* is the fundamental variable to be modeled with GMANOVA. It has no physical dimension and is somewhat difficult to interpret: For given *b* and *r*_1_, *Ei*[–*b*· *r*_1_] is constant, causing the growth curves to go through a given (calibrating) tree trunk radius *r*_1_. Therefore, for interpretation we can just look at the integral term *Ei*[–*b*·*r*] as a function of the radius *r*. The integral represents the area under the curve of *Exp*[–*b*·*r*]/*r*. With negative *b*, i.e., a sigmoid growth curve of the radius with time, the function *Exp*[–*b*·*r*]/*r* decreases from infinity at *r* = 0, has a minimum at the turning point radius of a sigmoid curve (*TPR* = −1/*b*), and subsequently increases again towards infinity at *r* =  infinity (the derivative of *Exp*[–*b*·*r*]/*r* towards *r*, set equal to 0, results in *r* = −1/*b*). The turning point radius is where the (instantaneous) increment of the radius is largest. With increasing radius, *Ei*[–*b*·*r*] increases rapidly at first, slows down until it reaches the turning point, and subsequently increases ever faster. Recall from (1) that (*Ei*[–*b*·*r*] – *Ei*[–*b*·*r*_1_])/*A* adds the time needed to reach a certain radius, and *Ei*[–*b*·*r*] divided by the initial relative growth rate *A* has the dimension of time (years). Given an initial relative growth rate *A*, the term (*Ei*[–*b*·*r*] – *Ei*[–*b*· *r*_1_])/*A* develops the time it takes to get a certain radius *r*. With negative *b*, the time it takes to reach a certain increment is ever smaller, until reaching the growth curve's turning point radius, after which the time becomes ever larger again. In case of exponential growth (*b* = 0), the term *Exp*[–*b*·*r*]/*r* becomes 1/*r* and its integral over *r* becomes *Ln*[*r*]. Correspondingly, *Ln*[*r*/*r*_1_]/*A* evolves time as the inverse function of the exponential growth function of the radius.

In the linear form of (10), the 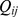 and 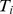 data can be used as input for the GMANOVA model, in order to calculate all 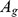 coefficients in a single model, rather than individually per tree. The GMANOVA model is used here for calculating linear regression through the origin for statistical comparison of several groups (sites), with residuals that may be heteroscedastic among time points. The origin corresponds to the calibrating point of the modeled mean growth curve for the group *g* (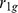 at 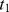), because 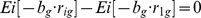 results in 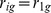. Note, however, that 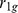 cannot be determined numerically from the regression with (10), because one would need to solve 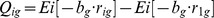 for 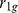, but the groups' 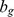 remain also unknown.

For the final regression it is recommended to delete the data column for *T* = 0. The regression goes necessarily through the origin, i.e., *Q* = 0 at *T* = 0. Therefore the data points at *T* = 0 have no influence on the results, cannot be transformed in a meaningful way (see the section on transformation below), and may distort the analysis of residuals.

When estimating first with (1) for each tree individually 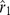 (and potentially also 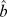, though not here), and use it subsequently for calculating 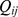 in (10), the errors 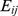 remain independent of the independent variable 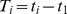. We therefore do not need to address the issue of estimation in two stages (two-stage least squares method or 2SLS; see chapter 9 in [25]). Rather, 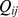 is a composite term with an error that is due to model inexactness as well as measurement and estimation error of 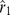.

We can use (10) for calculating variance components of the variation of the slope parameters *A* among groups, among individuals, and among repeated measurements. Developing a matrix with the same dimensions as ***Q***, the new elements are:
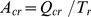
(11)


Here, 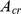 is the corresponding initial relative growth of data point 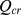 in column *c* and row *r*, and 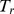 is the corresponding time value of row *r*. If the data was transformed, then the transformed 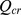 and 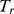 should be used, in order to allow the interpretation of slope data being distributed symmetrically around arithmetic means. The conversion of a data point *Q* at *T* with (11) can be thought of as drawing a line from the origin to each data point, and calculating the slope.

Note that the estimation of variance components carried out in this way does not depend on the regression slopes 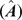 from the GMANOVA model, and can be calculated apart from the GMANOVA. Following pages 279-286 in [26], we define the model for calculating variance components as 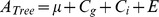, where 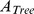 is the initial relative growth of a given tree, 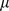 represents the parametric mean of the initial relative growth values 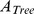 of all trees, 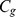 is a random contribution for the group (site) to the initial relative growth of a given tree, 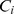 is a random contribution for the individual (tree) of a given site, and *E* is the error term, resulting from repeating the measurements at different times. 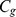, 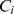, and *E* are assumed to have each a mean of zero, and the variances 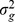, 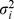, and 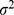, respectively. See pages 281–283 in [26] for the calculations, where 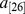 is here *k* (the number of sites), 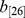 = *n*/*k* (the average number of trees per site), and 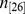 = *p* (the number of measurements over time). The expected mean squares are consequently 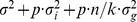 for sites, 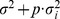 for trees, and 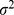 for time. It is assumed that there is no covariance among sites, trees, and time, and that there is no varying effect of time on trees. We are using here the procedure for a completely balanced design, even though our design with 16–27 trees per site is unbalanced on the intermediate level (being converted in the procedure here to an average number of trees per site). This could cause some bias in the estimates. We therefore verified our simple-to-calculate estimates with the more complex algorithms of the VARCOMP procedure in SAS (Statistical Analysis System). It turned out that our estimates for the variance components were in between the maximum likelihood estimates and the MIVQUE estimates from the SAS software.

### Data Transformation for the PL-GMANOVA Model

Ideally, one would like to have normally distributed *Q* values at each *T* value, and the residuals separately for each group (site) should at least be symmetrically distributed. With a total of 2,220 data points, each subgroup has on average 22.2 data points, pertaining to one of 10 groups and 30 time points. If the *data* of each such subgroup is at least symmetrically distributed, and a linear relationship over time is the right model, then the *residuals* will be symmetrically distributed. Therefore, we transform the data without subsequently analyzing the distribution of the residuals.

There is some trade-off between eliminating skewness and eliminating kurtosis, but focusing only on skewness, the both-sides power transformation is very effective to eliminate any level of skewness of a distribution, and maintains the linear relationship between time *T* and quantity *Q* in (10). Skewness is the major concern, because it causes bias of the mean growth path; kurtosis may affect “only” the statistical inference. Note that there are two different definitions of skewness and kurtosis in use: Pearson's “
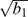
” and “
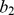
”, and Fisher's “
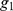
“ and “
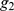
”; for formulas and conversion into each other see [27]. In a normal distribution, 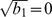, 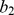 = 3, 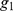 = 0, and 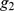 = 0. We used Pearson's 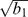 in our algorithm below.

A limitation for our application is that not only the *Q* values have to be transformed, but also the *T* values, and the GMANOVA model does not allow different *T* values for different groups. This in turn implies that one cannot use 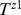 for one group, and 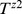 for another; only a global transformation can be applied to all groups, and consequently skewness cannot be eliminated completely for all groups, as different groups would require different power transformation. Nevertheless, in our case a global transformation remedies substantially non-normality.

Following, the separate treatment of negative *T* and *Q* is necessary to allow for negative non-integer numbers as *z* in the power transformation, without creating complex numbers. From (10) we get then:
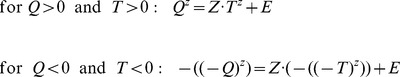
(12a – b)


Here, *z* is the power exponent that results from a systematic search to eliminate skewness within groups and time points; *Z* is the estimated regression coefficient that results with the transformed data; and *E* is the error term for the transformed data. In our application, the data points are almost only in the quadrants with both positive *T* and *Q*, or both negative *T* and *Q*. The only exception can be groups of data points that are typically found close to the origin. When used for an analysis with transformed data, these groups cause problems as influential outliers, because the positive data points move upwards, while the negative data points move downwards. To understand why this situation occurs close to the origin, consider the term of the *Y*-axis from (10), 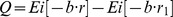: The term is negative for given *b* and radius *r* when 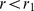 (going to minus infinity at zero and plus infinity when *r* goes to infinity). If the data points for a negative time interval *T* will result in negative *Q*, and the data points for positive *T* in positive *Q*, depends on *b* and *r*_1_ of the given tree, as well as how far *r* is away from *r*_1_ (which in turn depends on *t*).

We applied the following remedy, which keeps the problematic data points at a distance from the group's mean that is constant in terms of standard errors, and achieves that all data points are either in quadrants I or III after transformation:

1. Calculate the means and standard errors of all the *Q* in the groups, defined by given *T*, with mixed positive and negative *Q*.
2. Calculate in each group the distance from the mean in terms of standard error units of the opposite data points (positive *Q* at negative *T*, i.e., found in the upper left quadrant II; negative *Q* at positive *T*, i.e., found in the lower right quadrant IV).
3. Transform the data points with negative *Q* at positive *T*, as well as the data points with positive *Q* at negative *T*. Calculate the new means and standard errors in each group.
4. Calculate the distance of the opposite data points in terms of the new standard errors from the new means.

The procedure can be summarized in a formula that applies for the quantities at a given group and time point (e.g., for the Malacatépetl site the 24 quantities at *T* = −15):
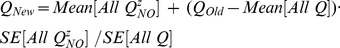



Here, 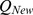 is the new quantity of the opposite data point after transformation; *Mean* is the arithmetic mean; 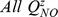 are the quantities of all non-opposite, transformed data points in the group at the time point 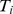; 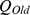 is the quantity of the opposite data point to be transformed; *All Q* are the quantities of all data points in the group at the time point 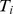; and *SE* is the sample standard error of *Mean*. In addition to its effectiveness to eliminate skewness, the advantage of the both-sides power transformation in cases of a linear regression through the origin is that *Z* can easily be back-transformed:
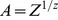
(13)


This results from putting *Q* = *A*·*T* into 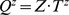, and solving for *A*. It shows also the important advantage that the linear relationship between *T* and *Q* is conserved, as the back-transformation to *A* in (13) does not depend on *T* or *Q*, while on the transformed scale the slopes are corrected for a better distribution of the residuals. Consequently, the idea of carrying out a linear regression, in order to get the slope parameters, is not affected by the transformation. To find the globally best *z*, we used the following algorithm:

- Convert the negative *Q* values of quadrant III to positive values by taking their absolute value. This is necessary to capture the positive skewness of the data in quadrant I as well as the negative skewness in quadrant III, when joining the data. Once the exponent for the power transformation is found for the joined absolute data values, (12b) is used to convert the formerly negative values again into negative values after transformation.
- For each group and each time point calculate the skewness from 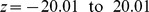, in steps of 0.5, resulting in 81 skewness values. Find the preliminary 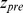, the *z*-value with the skewness being closest to zero. The shift of 0.01 avoids trying *z* = 0, which would result in 1 of all values and is non-sensical here.
- For a range from 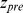 – 5.0001 to 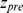 + 5.0001 calculate again the skewness on a finer scale in steps of 0.005. This results in 201 skewness values. Again, find the *z* with the skewness being closest to zero. This *z* is taken as the optimal one for the time point within the group.
- Gather all optimal *z*: For 10 groups and 30 time points, there will be 300 optimal *z*. Use the median *z* as the global one.

Confidence and comparison limits have to be calculated on the transformed scale, because they imply a normal distribution of the residuals. The numerical values of the limits can subsequently be back-transformed, resulting in confidence or comparison intervals on the original scale. The standard errors cannot be back-transformed, and thus have to be calculated on the original, untransformed scale, by using the original input data: Employing (13), with 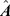, ***T***, and ***Q*** on the original scale, one re-calculates 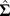 with (4) and subsequently 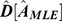 with (5) and 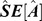 with (6). Standard errors derived in this way should not be used to calculate confidence or comparison intervals, and if one does, they will not coincide with the back-transformed intervals, because on the original scale the residuals are not distributed normally anymore.

### Calculation of Comparison Intervals for the Initial Relative Growth Estimates

Comparison intervals for the initial relative growth estimates 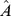 of the *k* groups can be calculated according to [28] (see also page 262 in [26]). If the data was transformed (like here), comparison intervals have to be calculated for the estimates from the transformed data (called 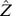). They can subsequently be back-transformed, as explained below, with (13):
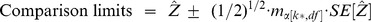



Here, 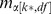 is the critical value of the studentized maximum modulus; 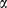 is the experiment-wide type I error rate (here 0.05); *k* ^*^ =  (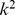-*k*)/2 = *k*!/(*k*-2)!/2, the number of pairwise comparisons among *k* groups (here with *k* = 10, *k* ^*^ = 45); 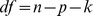, the degrees of freedom of all groups together; and *SE* =  standard error for the group in question from (6). While Miller [29] still hoped for more extensive tabled values of 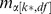, we calculated 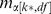 without any problem with *Mathematica*. The cumulative distribution function (*CDF*) of the studentized maximum modulus distribution in its correct form is difficult to find in the literature (here adapted from page 75 in [29]):
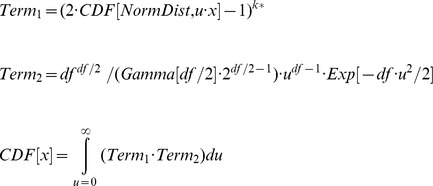



The cumulative distribution function 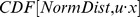 gives the probability value of the normal distribution (with mean  = 0 and standard deviation  = 1) that results from a value of *u*·*x*; *Gamma* is the gamma function. The integral has to be taken numerically, and there is no closed form of the inverse cumulative distribution function. Therefore, one has to vary *x* until *CDF*(*x*) equals 1-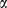, i.e., here *CDF*(*x*)  = 0.95. The resulting *x* is 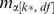. In our case, 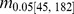 is 3.2983. Note that the integral has not to be taken to infinity, but only until there is no change anymore in the desired accuracy. Indeed, in our case an upper bound of *u* = 1.3 was already sufficient to stabilize *x* = 3.2983. The value was confirmed as intermediate of tabled values in [30].

### Field Data Collection and Processing for an Application of the PL-GMANOVA model

Trees were measured by the second author for his doctoral thesis [31]. The tree species *Pinus montezumae* A.B. Lambert (“Montezuma pine”) was found on ten sites at eight Mexican volcanos, at approximately 3,100 m above sea level and each about 500 times 500 m in size. The trees of this species grow up to 30 m in height, with a trunk diameter of up to 1 m. This pine species is widely distributed in Mexico and also in Guatemala, and not endangered. Here, the study sites were distributed in an area of 96 km×61 km (for details see Table 1). Herbarium specimens and core samples of 5 mm diameter were taken non-destructively from the trunks of the trees, after the authorities of the communal land had given their consent (in the case of the Popocatépetl site the office of the national park). In addition, the SEMARNAT (i.e., the federal environmental agency) had extended a permit to collect plant samples for scientific purposes.

**Table 1:** Volcano sites with 235 *Pinus montezumae* trees for dendrochronological analysis.

Volcano site		UTM X (m)	UTM Y (m)	Number of trees per site	Per-tree age in 2004 (years)	Per-tree radius in 2004 (cm)	Per-tree average annual increment (cm)
Catedral	Cat	450,104	2,168,173	25	43–84	18.7–36.8	0.08–0.67
Chichinautzin	Chi	482,041	2,109,907	26	48–139	9.5–19.3	0.04–0.16
Cuauhtzin	Cua	490,349	2,115,772	21	60–151	14.8–38.6	0.06–0.44
Guespalapa #1	Gue1	482,037	2,112,505	25	80–149	14.7–34.1	0.04–0.20
Guespalapa #2	Gue2	480,250	2,111,176	28	65–109	16.9–32.5	0.05–0.27
Malacatépetl	Mal	472,016	2,117,611	25	34–105	18.3–38.4	0.06–0.72
Pelado #1	Pel1	475,922	2,114,796	21	69–104	16.0–37.4	0.07–0.21
Pelado #2	Pel2	475,544	2,114,369	16	67–98	16.0–30.4	0.06–0.24
Popocatépetl	Pop	545,524	2,107,185	27	32–73	23.8–43.8	0.25–0.86
Tláloc	Tla	493,279	2,111,386	21	58–181	16.4–30.1	0.07–0.43
**Minimum:**		**450,104**	**2,107,185**	**16**	**32**	**9.5**	**0.04**
**Maximum:**		**545,524**	**2,168,173**	**28**	**181**	**43.8**	**0.86**

UTM zone 14, all sites approximately 3,100 m above sea level.

The sites represent landscapes with geologically different ages and corresponding different soil charateristics: The eight volcanoes are the Popocatépetl (1,000 years BP [ =  Before “Present” in geology  =  before 1950]), Chichinautzin (1,835 years BP), Guespalapa (two sites; 3,800 years BP), Tláloc (6,200 years BP), Cuauhtzin (8,000 years BP), Pelado (two sites; 10,000 years BP), Malacatépetl (30,500 years BP), and Catedral (100,000 years BP). The geological ages were estimates with ^14^C and K/Ar radiometric data.

Data collection and measurement followed standard dendrochronological procedures [32]. A total of 250 trees were measured, though only 235 trees at the end had data for 1974–2004 (16–28 trees per site). Two core samples per tree were taken at around 1 m trunk height with a Pressler increment borer of 5.15×40 cm, and the better sample of the two processed. Tree rings were cross-dated with a skeleton plot, spanning among all trees a range from 1824 to 2006. Core samples were mounted, polished, and measured on a movable stage under a stereozoom microscope (0.01 mm precision). The programs Verify5 (web.utk.edu/∼grissino/software.htm) and COFECHA (www.ncdc.noaa.gov/paleo/treering/cofecha/cofecha.html) were used to verify the measurements and the dating, respectively, of the 22,433 annual increments. Tree radiuses for the 30 years from 1974 to 2004 ranged from 9.5 to 43.8 cm, with per-tree average annual increments ranging from 0.04 to 0.86 cm. Tree ages varied between 32 and 181 years in 2004 (Table 1).

One way to calculate relative growth is to divide increment data by the corresponding intermediate quantity. For example, an annual radius increment of 0.3 cm divided by (10.1+10.4 cm)/2 is 0.029268 or 29.3% in a one-year period. A theoretically better formula [33] for calculating the mean growth rate is calculating (*Ln*[10.4] – *Ln*[10.1])/(1 year)  = *Ln*[10.4/10.1]  = 0.029270 = 29.3% again, when rounded. The formula is derived from the exponential function (10.1 cm)·*Exp*[(growth rate)·(1 year)]  = 10.4 cm. The difference is a constant growth rate that reflects “growth over growth” throughout the year, rather than assuming the relative growth at half the year to be representative for the whole year. The latter type of relative growth is employed here.

All statistical and mathematical calculations were carried out with several *Mathematica* 10.0 notebooks (www.wolfram.com), written by the first author (MR).

## Results from Applying the PL-GMANOVA Model

### Nonlinear Regression with the PL Model for Individual Trees

For each tree we used 31 data pairs of annual trunk radius (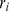) and the corresponding annual 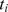 from 1 to 31 years (1974–2004), to apply the PL model with one segment individually. The nonlinear regression with (1) and 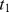 = 15 years resulted to be quick and stable with *Mathematica*, without problems of convergence. Table 2 summarizes the results. First, all three coefficients 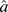, 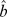, and 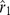 (with 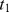 = 15 years in 1989) were determined by nonlinear regression (upper half of the table with “*b* free”). The complete set of parameters, represented in Table 2, consists of 1,880 parameters, corresponding to eight variables and 235 trees. For each of the eight variables the minimum, maximum, quartiles, and mean is given. The coefficient of determination ranges from 0.84 to 0.9998, and for half of the trees the fit is even between 0.9978 and 0.9993. As expected, the standardized residuals present statistically significant autocorrelation (“Probability of independent residuals” in Table 2). As mentioned in the methods, this autocorrelation is of no concern here for determining 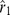, because the residuals are very small, relative to the corresponding radius (see Figure 1 top).

**Table 2:** Summary of parameters for growth curves (1974– 2004, *n* = 235 trees).

	Minimum	Maximum	Median	25%-quartile	75%-quartile	Mean
***b*** ** determined by regression**						
*R* ^2^	0.8351	0.9998	0.9988	0.9978	0.9993	0.9957
Probability of independent residuals	6·10^−18^	0.78	0.00013	4·10^−6^	0.0036	0.015
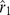 (cm)	4.57	37.53	21.52	17.41	25.20	21.37
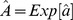 (year^−1^)	0.000015%	1.9·10^43^ %	16.43%	1.57%	189.27%	83.53%
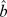 (cm^−1^)	−2.6544	0.6125	−0.1245	−0.2757	−0.0508	−0.2031
*corr* ( 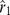 and 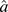 )	−1	1	0.746	0.727	0.762	0.732
*corr* ( 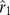 and 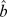 )	−0.99999	0.99998	−0.746	−0.762	−0.729	−0.733
*corr* ( 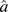 and 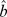 )	−0.99999	−0.9536	−0.9997	−0.9998	−0.9993	−0.9989
***b*** ** fixed according to the tree's midpoint radius**						
*R* ^2^	0.9563	0.9998	0.9967	0.9934	0.9984	0.9947
Probability of independent residuals	3·10^−16^	0.79	3·10^−8^	8·10^−11^	1·10^−5^	0.0075
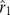 (cm)	8.25	37.50	21.54	17.50	25.31	21.68
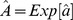 (year^−1^)	0.53%	21.33%	1.76%	1.26%	2.70%	1.92%
Fixed *b* (cm^−1^)	−0.1212	−0.0268	−0.0470	−0.0573	−0.0397	−0.0505
*corr* ( 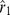 and 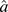 )	−4·10^−12^	1·10^−11^	−4·10^−16^	−4·10^−15^	1·10^−15^	2·10^−14^

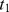
 = 15 years for all trees; corr  =  asymptotic correlation coefficients between regression coefficients; the probability for independent residuals is from a Ljung–Box test for randomness on the standardized residuals, with the null hypothesis that the autocorrelations 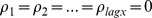 and alternatively that at least one 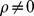.

The regression coefficient 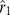 at 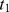 = 15 years varies widely from 4.6 to 37.5 cm among trees, corresponding to a wide range of the outer radiuses among trees (Table 1). On the other hand, the ranges of the regression coefficients 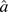 and 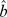 are exceedingly wide, for 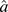 from −15.7 (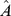 = 0.000015% per year initial relative growth) to 95.0 (1.9·10^43^ % per year), and for 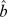 from −2.6544 (turning point radius  = 0.38 cm) to 0.6125 cm^−1^ (over-exponential growth). One would expect 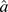 to fall into a range from approximately −8 to 0, and for 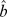 from approximately −0.5 to 0. An initial logarithmic relative growth of *a* = 0 represents 100% per year relative growth for the radius to start with (formally at a radius of 0 cm), and *b* = 0 cm^−1^ represents an exponential (instead of sigmoid) growth curve. The reason for the extreme parameter range here is caused by the rather linear growth curves in the 30-year segment. A straight line is determined by two coefficients, causing three coefficients in the nonlinear regression to be highly correlated. The range of the Pearson correlation coefficients between 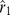 and 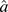, as well as 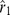 and 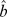, covers the whole possible range from −1 to 1. Furthermore, in the case of 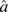 and 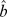 the correlation coefficients tend to be consistently close to −1.

To eliminate the correlation among regression coefficients for this data, *b* was fixed for each tree individually, such that the turning point radius (of a sigmoid curve) was at its midpoint radius, i.e., 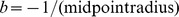. As can be seen at the bottom of Table 2 (“*b* fixed according to midpoint radius”), the remaining possible correlations between 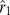 and 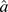 are basically zero. All three coefficients are now within reasonable ranges: Depending on the tree, the regression coefficient 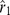 ranges from 8.25 cm to 37.50 cm, 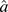 from −5.2 (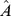 = 0.53% per year) to −1.5 (21.33% per year), and the fixed *b* from −0.121 to −0.027 (i.e., the turning point radius from 8.3 to 37.0 cm).

A further simplification (not carried out here) could be to fix not only *b*, but *b* and 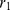 as *b*·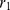 = −1. If one calculates the estimated 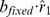 for each of the 235 trees, the range is from −1.1172 to −0.8170, and the mean is −1.0096 (the median −1.0056). This shows that in a segment of time as a function of the radius, where no curvature has to be taken into account, the growth curve can be symmetrically developed around the (in that case hardly recognizable) turning point radius *TPR* = 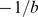, which then coincides with 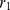. Therefore, one could set 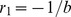, so that 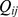 in (10) becomes 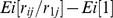, where 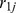 is the midpoint radius of tree *j*, and *Ei* [1]  = 1.89512. Consequently, if the segment in all trees is very close to a straight line, the nonlinear regression with (1) could be avoided altogether.

### Employing the PL-GMANOVA Model and Outlier Detection

Turning to the GMANOVA, the parameters of 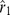 and fixed *b* for 235 trees were used to calculate 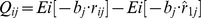 for each of the 235 trees as input for (10). In addition, with 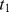 = 15 years, the 31 time values 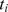 were converted into 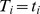 – 15 years. This is the data that entered into the GMANOVA. Going through the mathematical steps described in the methods, one gets the values for 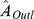 (“Outl” for inclusion of outliers) given in the third column of Table 3, together with the standard errors taken from the diagonal of the matrix 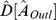.

**Table 3:** Initial relative growth with LSE and including outliers (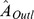), without transformation), and with MLE and excluding outliers (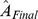, after back-transformation).

Volcano site	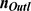	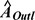 (year^−1^)	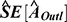 (year^−1^)	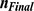	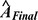 (year^−1^)	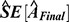 (year^−1^)	Probability 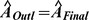	95% confidence limits of 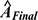 (year^−1^)
Cat	25	2.70%	0.404%	23	**1.73%**	0.154%	3.4·10^−14^	1.51–1.97%
Chi	26	1.78%	0.397%	25	**1.49%**	0.147%	0.0014	1.31–1.70%
Cua	21	1.88%	0.441%	20	**1.30%**	0.165%	2.1·10^−6^	1.11–1.51%
Gues1	25	1.39%	0.404%	23	**0.94%**	0.154%	8.2·10^−6^	0.81–1.10%
Gues2	28	1.64%	0.382%	27	**1.22%**	0.142%	2.3·10^−6^	1.07–1.39%
Mal	25	2.27%	0.404%	24	**1.19%**	0.150%	4.4·10^−16^	1.04–1.37%
Pel1	21	1.56%	0.441%	19	**1.18%**	0.169%	0.0013	1.00–1.38%
Pel2	16	1.63%	0.506%	16	**1.27%**	0.184%	0.011	1.06–1.50%
Pop	27	6.25%	0.389%	25	**3.72%**	0.147%	0	3.34–4.13%
Tla	21	2.36%	0.441%	20	**1.81%**	0.165%	7.0·10^−6^	1.57–2.09%

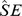
 =  estimated standard error; probabilities calculated with a t-test according to page 224 in [26]; confidence limits are from back-transformation.

**Figure 2:**
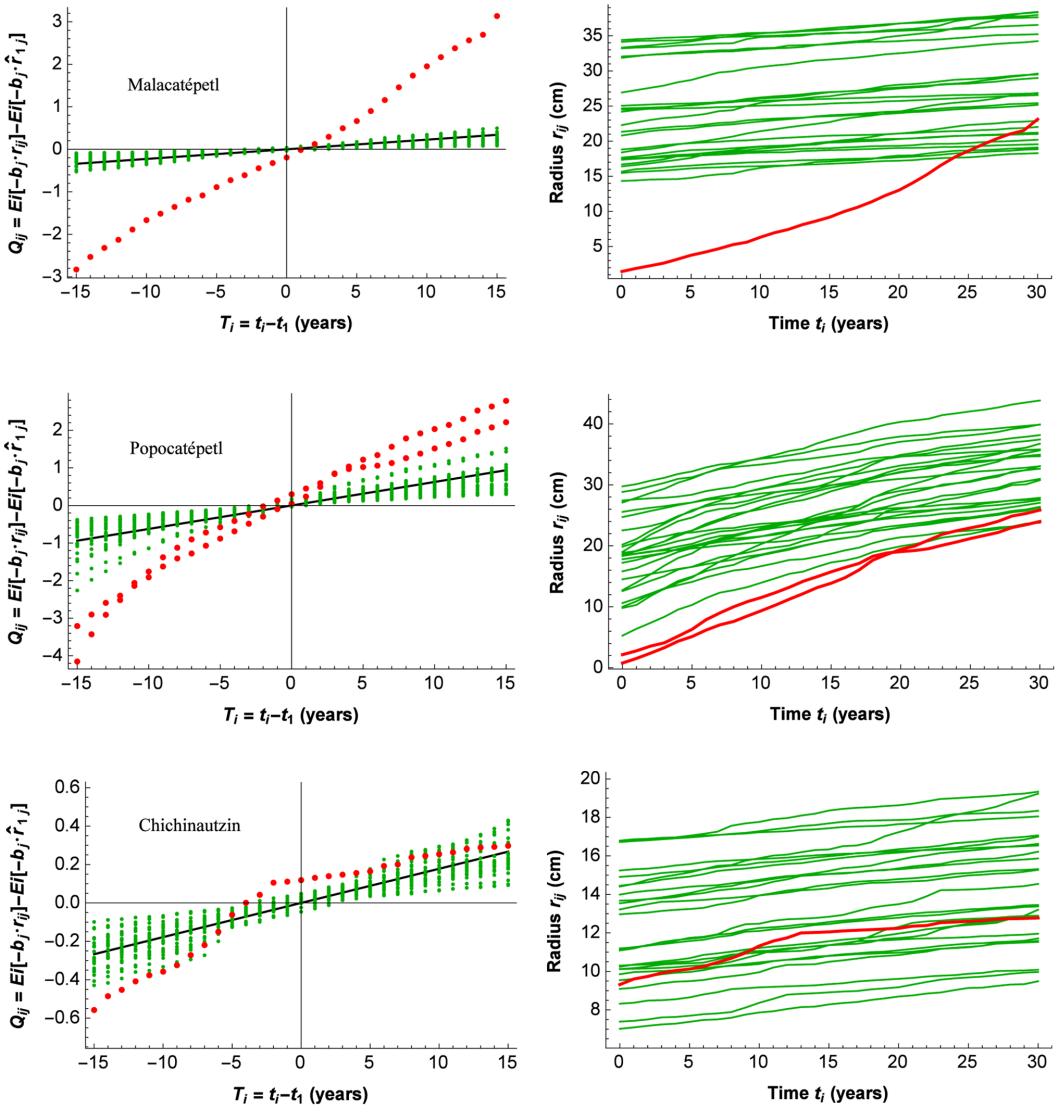
Outlier detection with the PL-GMANOVA model. On the left GMANOVA graphs for three sites, that represent initial relative growth 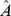 as the slope of the regression line with the least squares estimator (LSE), as well as the data points for time *i* and tree *j*. Outlying data curves, defined as data curves of which some points are at least four standard deviations from the regression line, are marked with red points. On the right, the original data curves with the outlying curves from the left marked again in red. Observe the masked outlying curve on the bottom right.

Figure 2 presents on the left the data points of 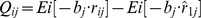 for three sites, with fixed *b*, as a function of the time points 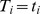 – 15 years. The straight line in black in each graph presents the regression function, calculated with the least squares estimator from (3), whose slopes are 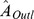 for the corresponding site. Outlying data curves are shown with red points (“data curves” represent the raw data, to be distinguished from the modeled growth curves). Following page 108 in [34], outlying data points were defined here as those points that are at least four standardized residuals away from the regression line. Standardized residuals were determined from the raw residuals 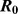 (see (7)), after calculating the leverages and the standard errors of the residuals of each group (page 525 in [26]).

Independently if one, several, or all data points of a tree's data curve were outliers, we defined the whole data curve as an outlying data curve. Such an outlying data curve is particularly obvious for the Malacatépetl site (Figure 2 top left). Not all data points of the outlying data curve for the Chichinautzin site are individually outliers (Figure 2 bottom left). The graphs on the right of Figure 2 present the original tree growth curves of radius as a function of time. The red points of the graphs on the left are red lines on the right. The key aspect that is shown here is that the outlying curves in the graphs on the left are very obvious, referring to an exceptional growth rate, while on the right that aspect can be masked. In the case of the Malacatépetl site, the outlying case is a younger tree of 34 years, with much faster growth, the median age for the site being 86.5 years in 2004; the curvature can easily be distinguished from the remaining tree growth curves (shown in green). The faster growth rate is not as easily distinguished in the graph for the Popocatépetl site (Figure 2 middle right). Note that the outlying aspect is not that the curves are at the lower bound of the set of curves, which rather indicates again younger trees with smaller radius (which tend to grow also faster), but the steeper curvature. Finally, for the Chichinautzin site (Figure 2 bottom) the outlying curve is completely masked in the right graph, being within the range of the radiuses of the other trees and not having a conspicuous curvature; actually, its growth rate is exceptional only during some periods of growth. In conclusion, the analysis shown on the left of Figure 2 provides an excellent tool to identify and distinguish exceptional growth, including when it occurs only during certain periods.

A total of 13 outliers were eliminated, 5.5% of the original 235 data curves. The data for all sites, except two (Gue1 and Mal), presented still considerable skewness after eliminating the outliers, and *z* = 0.2224 was determined as the globally best exponent for transformation. Since transformation cannot be carried out separately for the data of each site, when combining all groups in the GMANOVA model, the data for four sites remained significantly skewed (though less than originally), in particular for the Catedral (Cat) site. Kurtosis also improved in several cases, though not to the extent that the distribution of the data at each site could be considered normal. Nevertheless, the transformation fulfilled its purpose to get reasonable results with the maximum likelihood estimator from (2).

### Regression Coefficients and Comparison Intervals

**Figure 3:**
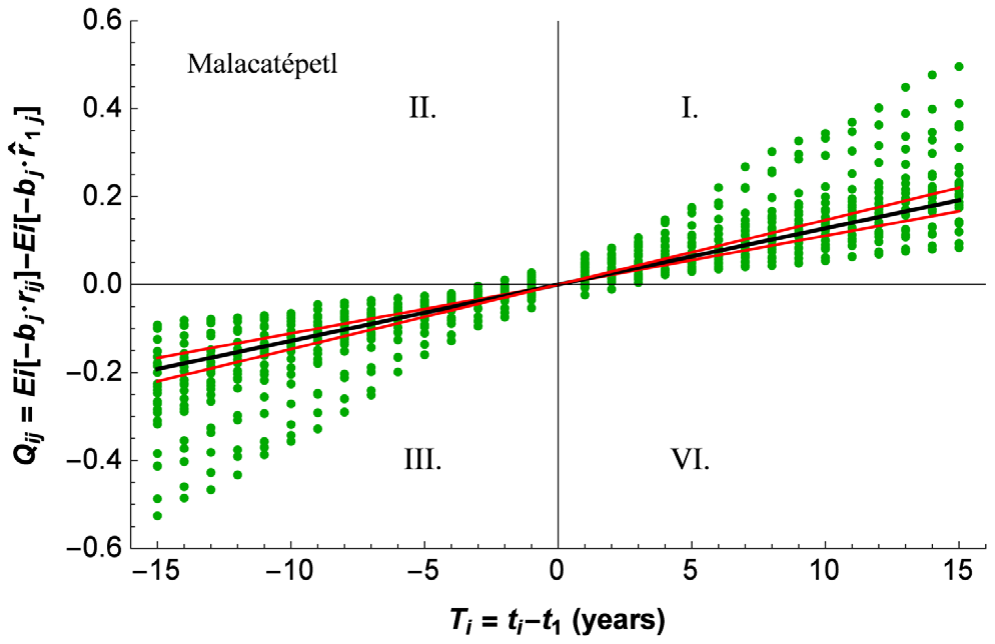
Data and regression line with the PL-GMANOVA model for the Malacatépetl site. The outlier shown in Figure 2 top was excluded. The data points for time *i* and tree *j*, as well as the regression slopes are back-transformed, causing slightly asymmetrical 95% confidence intervals. The regression slope is 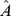 = 1.19% per year.

For the maximum likelihood estimation, the 16^th^ column for *T* = 0 years was deleted with the corresponding *Q* data (as in Figure 3), as recommended in the methods section of combining the PL and the GMANOVA models. Using 30 time points (from −15 to 15 years annually, without 0 years), the sum-of-squares matrix ***S*** had a determinant of 1.3·10^−46^ and a condition number of 41,358, the latter being the ratio of the largest to the smallest singular value (pages 50–55 in [35]). The inverse could nevertheless be calculated in the standard way.

Table 3 compares 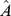, calculated first with the least squares estimator (LSE) with all the data, including the 13 outliers (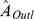), and subsequently the maximum likelihood estimator (MLE), excluding the data of outlying growth curves (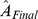). The number of growth curves (i.e., trees) in each case are given (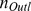 and 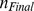). The initial growth rate 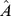 is significantly different for half the number of sites, when excluding outlying data curves. Furthermore, 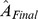 is always smaller than 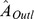, in a range from 16% to 47% per year, because (apart from different estimation techniques) the outlying data curves were frequently exceptionally fast-growing trees and not exceptionally slow-growing trees. The corresponding standard errors also become much smaller (62 to 64%). Note that for one site (Pel2), with no outliers deleted (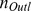 = 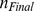 = 16), the parameters nevertheless change. This is due to using different estimators (LSE and MLE) and having improved the distribution of the data with a transformation for maximum likelihood estimation.

Figure 3 shows on the bottom the back-transformed data with regression lines for one site (Malacatépetl  = Mal). The non-normally distributed residuals are obvious, as well as the reasonable positioning of the regression lines with the maximum likelihood estimator. The slope of the intermediate regression line is numerically equal to 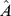 = 1.19% per year). The other two regression lines represent slightly asymmetrical 95% confidence limits.

Having estimated the mean initial relative growth 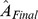 for each site, can we draw out a new growth curve with these estimates? Yes, but we would have to decide still about some unknown parameters: For employing 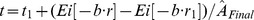 from (1), we would need the parameters for 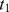, *b*, and 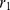, in addition to 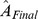. One way to solve this is to decide that the growth curve should start at the measured median radius in 1974 (*t* = 0 years) and finish at the median radius in 2004 (30 years). Taking the Malacatépetl trees of Figure 3, the median growth curve should then go from 21.3 cm and finish with 26.5 cm. Therefore one gets two equations with two variables: 0 = 15+ (*Ei*[–*b*·21.3] – *Ei*[–*b*·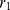])/0.0119 and 30 = 15+(*Ei*[–*b*·26.5] – *Ei*[–*b*·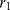])/0.0119. This system of two nonlinear equations can be solved (e.g., in *Mathematica* with the FindRoot function). The result is *b* = −0.0206 cm^−1^ and 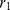 = 23.8 cm. The objective here, however, was to compare mean relative growth among sites in a standardized way, not to predict a median growth curve for each site.

**Figure 4:**
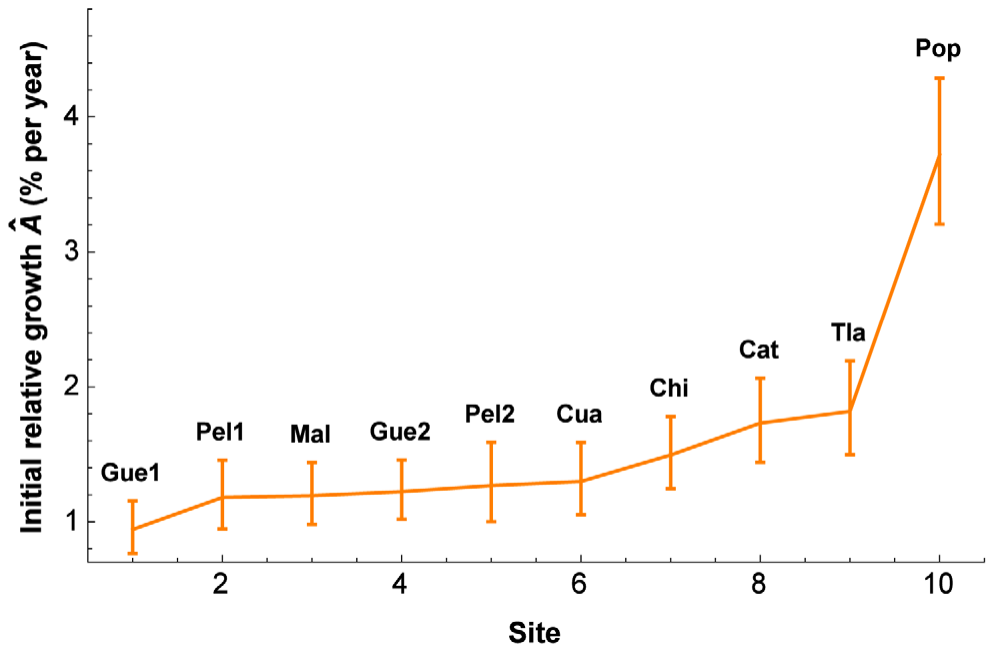
Comparison intervals of initial relative growth (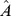 **) for the trees on each sites.** Means of non-overlapping 95% comparison intervals are considered to be significantly different. The comparison intervals are back-transformed, and consequently slightly asymmetrical. The sites are ordered according to increasing 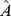. See Table 1 for site abbreviations and site information. The trees on the Popocatépetl site present a significantly higher mean 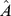 than all other sites. In addition, the following pairwise comparisons indicate significant differences: Gue1-Chi, Gue1- Cat, Gue1-Tla, Pel1-Tla, Mal-Cat, Mal-Tla, and Gue2-Tla.

Figure 4 presents 95% comparison intervals for the ten sites. Comparison intervals were calculated on the transformed scale, and then back-transformed with (13). One can observe some slight asymmetry around the mean values, especially in the case of the Popocatépetl. If a comparison interval does not overlap between two sites, then the two sites are considered significantly different. The distinctively higher growth rate on the Popocatépetl site (Pop) is especially obvious. Initial relative growth of the trees on the Popocatépetl is 3.9-times the one of the trees on the Guespalapa #1 site (Gue1), and significantly higher than that on all other sites. Apart from the Popocatépetl site, there is a continuous increase from the Guespalapa #1 site to the Tláloc (Tla) site, with seven pairwise comparisons indicating significant differences (see legend of Figure 4).

### Model Fit and Variance Components

The coefficient of determination from (8) is 0.986, which indicates that a high proportion of the data's variance is explained by the model. For evaluating the model fit further, *Median*[*All Abs* 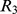]/*Median*[*All Abs* (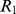
+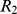)] was calculated, as explained in the methods section after (9). The ratio is 0.53, indicating that data deviation from the model can be considered sufficiently smaller than growth variation due to nature's within-site factors or measurement error. A Mann-Whitney *U*-tests comparing the medians is highly significant. In that sense, also the significant autocorrelation of the means of *Q* among the time points for each site is not a mayor concern; apparently it is caused by a necessarily imperfect global power transformation of the data points from all sites together.

Calculating the sample variances for each of the 10 groups at each of the 30 time points, we get a matrix with 300 variances. Variances may me heterogeneous among time points, but should be homogeneous among groups (within time points). When calculating the ratios of the largest variance to the smallest variance among the 10 groups, we get one ratio for each of the 30 time points. These ratios are on average 3.8, ranging from 2.4 to 4.8 (rather than the ideal 1). A Conover test for homogeneity of variances (pages 239–248 in [36]) is significant at 10 time points, and non-significant at 20 time points, i.e., the heteroscedasticity varies with time points, being significant in one third of them. The median probability is 0.14, the lowest 0.007. The heteroscedasticty among groups makes the standard errors of the estimates of *A* and the comparison intervals in Figure 4 less exact, but is not of mayor concern for our conclusions.

Calculating the ratios of the largest variance to the smallest variance among the 30 time points, we get one ratio for each of the 10 groups. These ratios are on average 4.3, ranging from 2.6 to 7.7. In a Conover test for homogeneity of variances, only one time point presents significant heteroscedasticity, but as mentioned the model allows heteroscedasticity among time points.

Calculating variance components for the initial relative growth (*A*), 34% of the growth variation was found among sites, 31% among trees, and 35% over time. Without the Popocatépetl site, the numbers changed to 7% among sites, 42% among trees, and 51% over time. For explaining more growth variation, further research should focus on environmental factors that cause variation within sites, such as soil, water availability, and competition, as well as factors that change over time, such as competition among trees. Genetic variation within the 16–28 trees per sites is less likely to play a major role, given their relatively small size of about 500 times 500 m, and it does not change with time.

## Discussion

The PL growth function from (1), 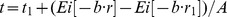 for 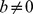 and 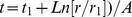 for 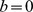, was originally derived in [4], and subsequently again applied in [37], based on the (possibly piecewise) linear relationship of logarithmic relative growth as a function of tree trunk diameter or radius. Originally the motivation was to determine a growth curve when time or age values are unknown in tropical trees. The resulting function, however, has some interesting mathematical properties, which motivated us to explore its use as a general growth curve function, including when time values are known:

1. The three coefficients *a*, *b*, and 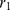 (at given 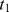) have straight-forward interpretations: *a* is initial logarithmic relative growth (i.e., the *Y*-intercept at *r* = 0), serving as a standardized growth rate parameter; if *b* is negative, then it is numerically the negative inverse of the turning point radius (from a left-winged to a right-winged growth curve), serving as a shape parameter; and 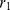 at 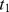 calibrates the position of the growth curve segment. Figure 1 illustrates this interpretation of the parameters in the case of one tree.
2. All types of monotonic growth of the tree radius (or another quantity) over time can be modeled in a very flexible way. Depending on *b*, the growth curve segment is sigmoid, exponential, or over-exponential (“exploding”). Depending on 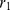 (or 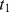) and *A* =  *Exp*[*a*], the curvature part that fits the data best is applied (left-winged, right-winged, or sigmoid); 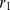 and 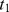 can also be outside of the measured range, and 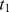 can even be negative. Finally, a large negative value of *a* can cause the growth curve to be virtually linear. Growth can either be positive or negative (however, not mixed or cyclical).

Employing the PL model together with the GMANOVA model provides some additional advantages:

1. Hidden outlying data curves are easily detectable, both numerically and visually, as was explained with Figure 2.
2. Differences of growth rates among groups (here trees on different volcano sites) can be quantified and compared statistically in a very efficient way that is statistically robust, takes into account different starting and turning point radiuses, and allows for heteroscedasticity of the residuals among time points.

Another approach would be to apply the PL model nonlinearly for individual trees with (1), as done here, but avoid the GMANOVA model. With the results summarized in Table 2 bottom (with fixed *b*), one could simply calculate the average and standard error for the trees per site. The estimate of 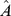 is larger for all sites, by a factor from 1.2 to 1.4 (with no transformation applied). The pooled variance of the GMANOVA is smaller by a factor of 2.2. This difference in variance comes as an advantage from analyzing all 222 trees together, rather than separately 22 trees for the Catedral site (Cat), 25 trees for the Chichinautzin site (Chi), and so on.

An even simpler way to compare tree growth is to compare annual increments. Calculating average annual increments, these range from 0.10 cm (Chi) to 0.46 cm (Pop). Ordering sites according to increasing average annual increment, the sequence differs for the annual increments compared with the sequence shown in Figure 4: Chi <Gue1<Gue2<Pel2<Pel1<Tla<Cua<Mal<Cat<Pop. The only site that does keep the same position is the Popocatépetl (Pop). In particular the Chichinautzin (Chi) tree group moves from the slowest-growing position in increment to the third-fastest position in initial relative growth. One reason is that the radiuses are smaller, and thus the growth rate (relative growth) is faster than revealed by analysis of the annual increments. The idea of using the PL-GMANOVA model is to compare growth on “equal footing”, as if the initial radiuses were the same.

If we were to employ a standard multiple linear regression model (without taking into account any degree of dependence or heterscedasticity of the residuals), we would *not* obtain our final results directly: Employing the regression model *Q* = *T*+ *Site* +*T*·*Site* +*E*, where *Site* is a dummy variable, one obtains one regression coefficient for *T*, ten coefficients for the sites, and nine coefficients for the interactions *T*·*Site*, each with its standard error.

Heteroscedasticity and autocorrelation of the residuals could be handled in a more flexible way by the mixed(-effects) linear model, as introduced by Laird and Ware [38]. That model has become popular in recent years [39]–[42]. The model is also called “hierarchical model”, among other terms, and indeed the different names cause confusion: The GMANOVA model itself is a special case of a linear mixed model, where the design matrix of fixed effects is a linear combination of the design matrix of random effects (page 184 in [43]). We therefore talk about “Laird's (linear) mixed model” for distinction.

The algorithm of Laird's mixed model is iterative and complicated [44], [45]. Convergence problems are reported in the literature (page 109 in [46]; they mention 2% of their samples under default options). Such problems are avoided by the GMANOVA model with its closed-form equations. While generally the estimators can be unbiased, model verifications and inference in Laird's mixed model are based on asymptotic statistics (when the number of observation goes towards infinity). Formulas for exact standard errors or confidence intervals, and thus for exact statistical inference, have not been found (see page 188 in [40], pages 166–173 in [42]), page 92 in [44]).

An advantage of Laird's mixed models is the easy handling of missing and unbalanced data, i.e., data where individuals have different numbers of repeated measurements, possibly obtained on different occasions (page 22 in [41]). There are methods for the GMANOVA model to handle randomly missing or incomplete data, but not observations at irregularly spaced intervals or non-randomly missing data (page 235 in [47]). This is, however, in general not a problem with dendrochronological data, as long as one chooses a common interval of measured years. Randomly missing data values for one year can easily be interpolated linearly from the data values of the adjacent years. Combining longer with shorter time series, however, causes not only computational issue, but also conceptual questions about what the longer time series can tell us about the shorter time series in the period that the latter does not cover.

After having quantified the differences among mean tree growth curves on the ten sites from eight volcanoes, how can we explain the detected differences, in particular the much faster tree growth on the Popocatépetl site? There could be much more favorable site conditions, such as for soil nutrients and water availability. Among sites, it is also more likely to find genetic differences. One different aspect, however, needs to be mentioned here that has caused several outliers in Figure 2. If a period between two dates is used (as here from 1974–2004) and trees do not have the same age, then the growth curve comparisons among trees represent the same exposition to climate, but differ in age-specific growth phases. On the other hand, if tree age is used, the age-specific growth phases are the same, but the influence of climate varies among trees. With a mixed-age population of investigated trees, it is impossible to have neither the varying-age nor the climate effect, so one has to choose what effect is to be controlled. Even when adjusting 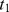 in (1) individually for initial age of each tree, we do get the same parameters, because the model is based on a growth difference during a time period 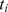 – 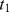. Our model took differences in the initial radiuses into account for comparing growth, but not that trees of the same radius, but different age, do likely imply different growth physiology. Tree ages in 2004 varied for the 222 trees from 36 to 151 years; the 25% and 75% quartiles are 68.75 and 98 years, respectively. Indeed, the youngest trees of the study are at the Popocatépetl site (Table 1, median age  = 54 years). If we compared growth at the same ages, however, we would compare with potentially different influence of climate with its strong impact on growth. While tree age is unlikely to explain the much faster growth at the Popocatépetl site by itself, it would be desirable to include initial tree age (in 1974) as an explanatory variable in our model. Such a model adaptation, to include explanatory variables, will be the topic of a future contribution.
